# Congenital Absence of the Portal Vein as a Rare Cause of Portopulmonary Hypertension—A Case Study Series

**DOI:** 10.3390/medicina58101484

**Published:** 2022-10-19

**Authors:** Tereza Hlavata, Monika Kaldararova, Filip Klauco, Erika Drangova, Adriana Reptova, Iveta Simkova

**Affiliations:** 1 Department of Cardiology and Angiology, Adult Congenital Heart Disease Department, Medical Faculty, Slovak Medical University and The National Institute of Cardiovascular Diseases, Pod Krasnou Horkou 1, 833 48 Bratislava, Slovakia; 2 Department of Diagnostic and Interventional Radiology, Medical Faculty, Slovak Medical University and The National Institute of Cardiovascular Diseases, Pod Krasnou Horkou 1, 833 48 Bratislava, Slovakia

**Keywords:** congenital absence of the portal vein, congenital portosystemic shunt, pulmonary arterial hypertension, portopulmonary hypertension, specific therapy, pulmonary artery aneurysm

## Abstract

*Background*. Congenital absence of the portal vein (CAPV) is an extremely rare malformation that is caused by aberrant venous development during embryogenesis and is usually associated with congenital portosystemic shunts (CPSS). This hemodynamic allows mesenteric blood to bypass the liver metabolism and causes an imbalance between vasodilators and vasoconstrictors in the pulmonary circulation, which, again, might lead to the development of secondary portopulmonary hypertension (PoPH). Establishing the exact morphology of the splanchnic venous system is important when evaluating possible therapeutic options (differentiating type I and II CAPV), because some variants enable the closure of the shunt, and this represents a potential cure for pulmonary arterial hypertension (PAH). Once PoPH is diagnosed, complex care in a specialized expert centre is necessary. If possible, CPSS closure is recommended. For long-term successful patient management, specific targeted PAH therapy administration is crucial. Significant morbidity and mortality in these patients may result not only from PAH itself but also due to specific PoPH complications, such as compression of the left main coronary artery by pulmonary artery aneurysm. *Case Report*. We report on two patients with PoPH due to CAPV and CPSS (without any liver disease) who presented as severe PAH and who, before admission to our expert centre, were misdiagnosed as idiopathic PAH. The case reports also represent our experience with respect to the long-term follow-up and PAH-specific medical treatment of these patients, as well as the possible (even fatal) complications of these rare and complex patients.

## 1. Introduction

Congenital absence of the portal vein (CAPV) is a rare malformation that is caused by aberrant venous development during embryogenesis. CAPV is usually also associated with the presence of a congenital portosystemic shunt (CPSS), where the blood carried by the superior mesenteric vein and the splenic vein bypasses the liver and is drained directly into the systemic venous circulation. This abnormal mesenteric drainage causes the loss of physiologic hepatic clearance, and this—again, due to a long-term significant imbalance in the vasoactive substances involved in normal pulmonary vascular integrity—may result in the development of secondary pulmonary arterial hypertension (PAH) [1,2].

The first case of CAPV was reported by John Abernethy in 1793 based on the post-mortem examination of a 10-month-old female, which showed the termination of the portal vein in the inferior vena cava at the insertion level of the renal veins and multiple other congenital anomalies, including dextrocardia, transposition of the great vessels and polysplenia [3]. “The Abernethy Malformation” was an eponym for congenital extrahepatic portocaval shunts.

**Figure 1:**
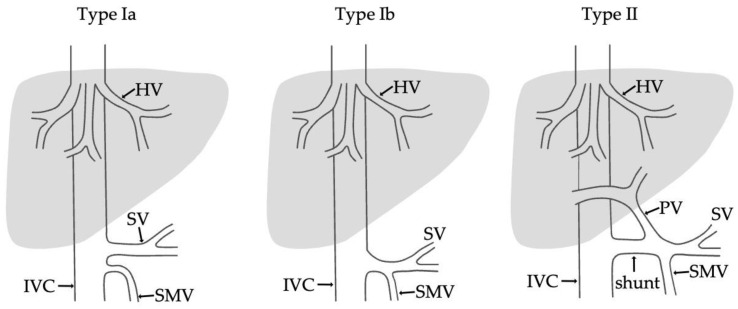
Classification of Abernethy malformation according to Morgan and Superina. IVC—inferior vena cava; SMV—superior mesenteric vein; SV—splenic vein; HV—hepatic veins; PV—portal vein.

Various classifications for the evaluation of extrahepatic portosystemic shunts have been proposed. The most practical and most frequently used in the literature is the classification by Morgan and Superina, who divided CPSS in two types based on whether or not the hepatic parenchyma is perfused with blood from the mesenteric venous system (Figure 1):

- Type I—end-to -side anastomosis with a complete portosystemic shunt exhibiting no visible portal flow in the liver due to the absence of intrahepatic portal veins. The malformation is female-predominant and associated with other congenital abnormalities, such as cardiac defects.
- Type II—side-to side-anastomosis with hypoplastic intrahepatic portal veins, and the liver is perfused with portal blood in the presence of a partial shunt (e.g., porto-hepatic venous anastomoses) [4].

Additionally, type I is subdivided into two further types depending on the anatomy of the portal vein. In type Ia, the splenic vein and the superior mesenteric vein drain separately into the inferior vena cava or the left renal vein, while in type Ib, a confluence of the superior mesenteric and splenic veins is present, but it does not supply the liver [5,6].

According to this classification, CAPV associated with extrahepatic portocaval shunts can be referred to as an Abernethy type I malformation. To provide an accurate assessment of the patency of the intrahepatic veins, an angiography with the occlusion of the shunt should be performed so as to confirm the assumed absence of intrahepatic veins or presence of remnant hypoplastic hepatic branches [7,8].

To our best knowledge, only 182 cases of patients with Abernethy type I malformations have previously been reported in the literature [9].

## 2. Case Presentation

### 2.1. Clinical Case 1

A 41-year-old male with a history of frequent respiratory infections during childhood was under long-term observation for idiopathic PAH. This diagnosis was established at the age of 4 by right heart catheterization (RHC), when the mean pulmonary artery pressure (mPAP) was 41 mmHg. Congenital heart defect was excluded, and pulmonary arteriopathy with medial hypertrophy and intimal thickening upon lung biopsy was histologically confirmed.

The patient was clinically stable for 19 years, with no dyspnoea during physical activities (New Your Heart Association (NYHA) class I). After this period, he was re-evaluated due to the occurrence of shortness of breath on exertion and significant fatigue. On an electrocardiogram (ECG), a previously documented incomplete right bundle branch block was observed, and transthoracic echocardiography showed preserved systolic function of the dilated right ventricle, without tricuspid regurgitation. An invasive haemodynamic assessment confirmed the presence of severe PAH (mPAP 59 mmHg), pulmonary capillary wedge pressure (PCWP) of 8 mmHg and pulmonary vascular resistance (PVR) of 7.2 Wood units (WU), with no vasoreactivity (Table 1). Due to the patient’s objectively demonstrated good physical performance (the patient achieved 8 METs on treadmill ergometry), a watchful, conservative approach without any treatment was selected, as specific therapy for PAH was not available at that time.

**Figure 2:**
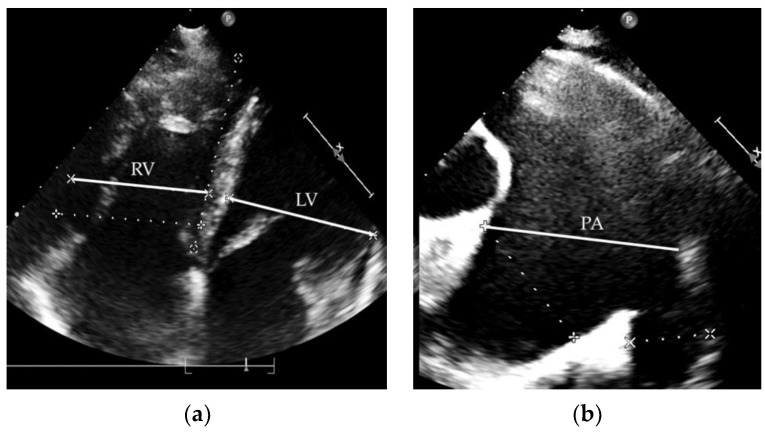
Patient 1 echocardiogram: (**a**) dilated right ventricle; (**b**) dilated pulmonary artery. RV—right ventricle; LV—left ventricle; PA—pulmonary artery.

During the next nine years, the further progression of dyspnoea and reduction in exercise capacity continued. At the age of 31, the patient was admitted to our department due to decreased physical function (NYHA class III), with peripheral oedema, severe hoarseness and abdominal discomfort. An abdominal ultrasound showed fibrotic liver remodelling without a nodular lesion and mild ascites. On Doppler imaging, blood flow was present only in the dilated hepatic veins, with no portal flow in the liver. Computed tomography confirmed the intrahepatic absence of the portal vein, with a confluence of the superior mesenteric vein and splenic vein, which were draining directly into the inferior vena cava through a CPSS. Other indirect signs suggestive of CPSS were a slightly higher level of ammonium in the blood and an electroencephalogram (EEG) pattern of encephalopathy. Throughout the years, no liver nodules were observed on ultrasound. Esophagogastroduodenoscopy showed no signs of portal hypertension (no oesophageal or gastric varices). The blood level of the liver enzymes was in the normal range. Echocardiography showed a dilated right ventricle (Figure 2a) with mildly decreased systolic function, mild tricuspid regurgitation with increased regurgitation pressure (80 mmHg) and a dilated pulmonary artery (Figure 2b). RHC confirmed the progression of PAH with increased pressure in the pulmonary artery (mPAP 90 mmHg, PCWP 10 mmHg, PVR 12.6 WU) (Table 1). The test of the pulmonary function revealed obstructive pulmonary disease with a significantly reduced carbon monoxide diffusing capacity.

In the light of these results, the diagnosis was reclassified as PoPH due to CAPV, with severe secondary PAH. The atypical clinical symptom, hoarseness, was diagnosed as a result of laryngeal nerve paresis due to the dilated pulmonary artery. According to the guidelines already available at that time, PAH-specific therapy with phosphodiesterase-5 inhibitor sildenafil was initiated with concomitant therapy for right heart failure, including diuretics and spironolactone.

During the further follow-up, clinical improvement was observed after the specific therapy had been started (Table 2). The patient demonstrated an increased 6 min walking test distance, and the patient’s dysphonia also improved as a result of the decreased pressure of pulmonary artery on the recurrent laryngeal nerve. This was confirmed by RHC, where decreased mPAP and PVR were measured, as shown in Table 1. Since there was no liver affection and the positive effect of the specific therapy was proven, liver transplantation was not recommended.

**Figure 3:**
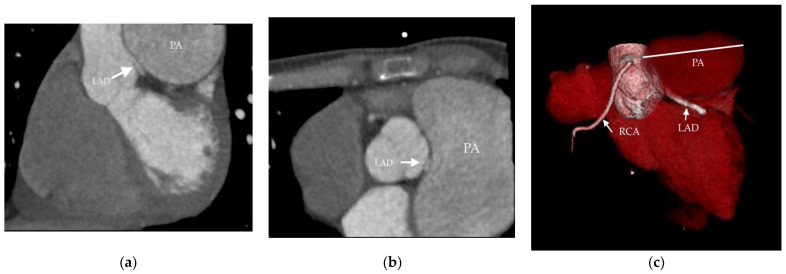
Coronary computed tomography angiography (Patient 1). The arrow shows the left ascending artery (LAD) compressed by dilated pulmonary artery (PA): (**a**) sagittal view; (**b**) axial view; (**c**) 3D reconstruction.

On the other hand, despite good clinical results, the dilatation of the pulmonary artery progressed. This was manifested by computed coronary angiogram, which revealed compression of the left main coronary artery (LMCA) and the proximal left anterior descending artery by severely dilated pulmonary artery (Figure 3). However, no symptoms of LMCA compression were present. The ECG was without ischemic changes, and patient had no chest pain, either resting or with exercise.

After 11 years of specific PAH treatment with a stable clinical condition, the patient was admitted to a regional hospital with a history of fever lasting for 3 days, shivering and a cough associated with angina. In the laboratory results, a high level of troponin and inflammatory markers (C-reactive protein, procalcitonin) were present. In the regional hospital, several paroxysms of ventricular tachycardia and ventricular fibrillation occurred, with a need for defibrillation.

**Figure 4:**
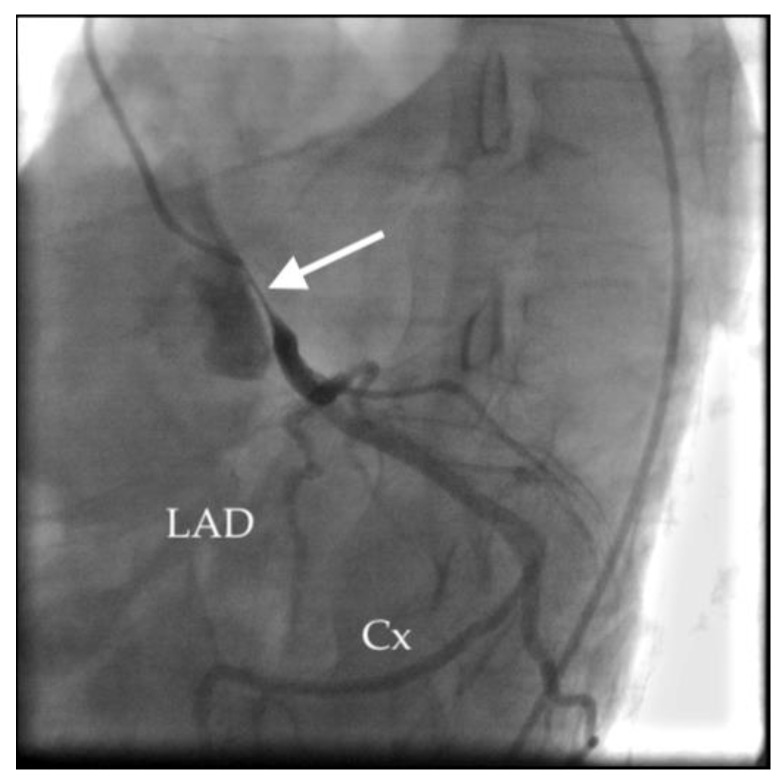
Patient 1 coronarography: arrow shows critical extrinsic compression of the left main coronary artery.

The patient was transferred to our heart centre on suspicion of acute coronary syndrome associated with severe pneumonia. Upon admission to the intensive care unit, he was orthopneic, cyanotic, hypotensive and on vasopressors and inotropic support, with a need for repeated electric cardioversion due to runs of ventricular tachycardia. Coronarography revealed critical ostial stenosis of the LMCA artery due to extraluminal compression by the extremely dilated pulmonary artery (Figure 4), without coronary atherosclerosis. Transcatheter coronary intervention on the LMCA was not possible due to the patient’s unfavourable coronary anatomy and haemodynamic instability. An acute myocardial revascularisation by coronary artery bypass grafting (right great saphenous vein to the left anterior descending artery and the left marginal artery) was performed. Despite this complex treatment, the patient died within the next 3 h after the surgery due to severe sepsis and cardiogenic shock.

### 2.2. Clinical Case 2

**Figure 5:**
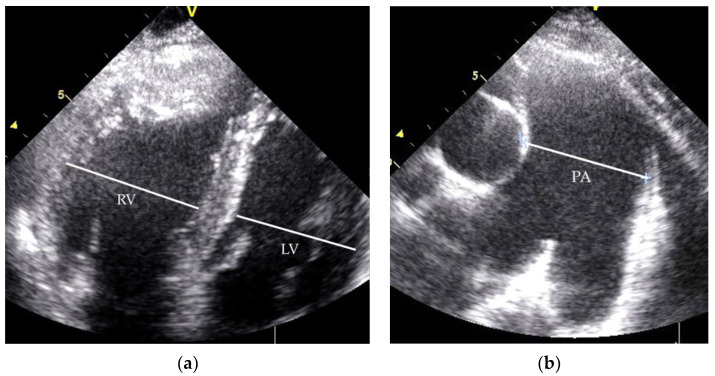
Patient 2 echocardiogram: (**a**) dilated right ventricle; (**b**) dilated pulmonary artery. RV—right ventricle; LV—left ventricle; PA—pulmonary artery.

A 44-year-old woman with a previous long-term history of splenomegaly and mild thrombocytopenia was first admitted to a regional hospital as a 26-year-old after several syncope episodes provoked by exertion. On physical examination, a mild diastolic murmur above the pulmonary valve and accentuated second heart sound were present. An echocardiogram revealed the D-shape of the left ventricle with ventricular sept flattering and an enlarged right ventricle (Figure 5a) with mild tricuspid regurgitation. The tricuspid regurgitation gradient was 65 mmHg. RHC showed severe PAH without vasoreactivity (mPAP 58 mmHg, PWCP 9 mmHg, PVR 8.5 WU), as presented in Table 3, with no signs of thromboembolic disease on pulmonary angiogram. After excluding any intracardiac shunt, the diagnosis of idiopathic PAH (“primary pulmonary hypertension”, according to the classification at that time) was established.

**Figure 6:**
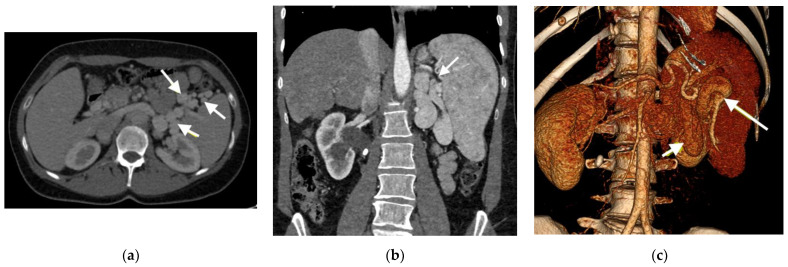
Computed tomography (Patient 2). Arrows show varicose dilatation of the splenic vein: (**a**) sagittal view; (**b**) coronal view; (**c**) 3D reconstruction.

During the next five-year follow-up period, the slow progression of dyspnoea and intermittently, mildly increased liver function, observed upon blood test, (bilirubin, aspartate aminotransferase, alanine aminotransferase), occurred, accompanied by discrete peripheral oedema. An abdominal ultrasound revealed massive fibrotic changes in the portal vein, with no blood flow, normal liver parenchyma and splenomegaly. The subsequent computed tomography (CT) angiography diagnosed the agenesis of the portal vein, with a huge portosystemic shunt from the dilated splenic vein to the left renal vein (Figure 6). No liver nodules or signs of portal hypertension were detected. Clinical signs of hepatic encephalopathy were not present. Spirometry showed normal lung function.

The diagnosis was re-classified as PoPH due to CAPV. The patient received consultation at a foreign surgical centre specializing in congenital vascular malformations and surgical reconstruction. Neither transcatheter shunt closure nor liver transplant were recommended. Afterwards, according to guidelines, PAH-specific therapy with selective treatment for the type A endothelin receptor antagonist (ERA), ambrisentan, was initiated.

**Figure 7:**
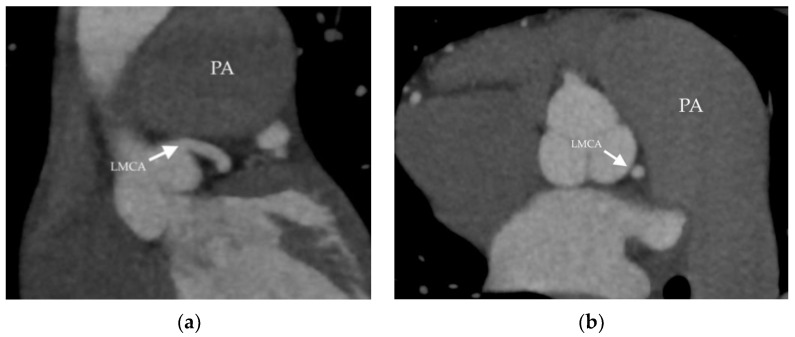
Coronary computed tomography angiography (Patient 2). The arrow points to the left main coronary artery (LMCA)- not compressed by the dilated pulmonary artery (PA): (**a**) sagittal view; (**b**) axial view.

Within the next 3 months, clinical improvement was demonstrated, with increased exercise capacity, NTproBNP normalization and a mild reduction in the size of the right ventricle and PAH (Table 4). This favourable clinical situation was sustained for the next 10 years. When the progression of the pulmonary artery dilatation was recorded (Figure 5b), coronary artery compression was excluded by CT coronarography (Figure 7).

During the further follow-up period, after 10 years of specific PAH treatment, a mild deterioration in the exercise capacity (NYHA II), elevation of the NTproBNP blood levels and progression of RV dilatation were observed (Table 4). A combined, dual specific treatment with phosphodiesterase-5 inhibitor sildenafil was initiated but was not well tolerated by the patient due to symptomatic hypotension. Currently, her clinical status is stable on monotherapy, with the option of exchanging ambrisentan for a more selective ERA macitentan if any further deterioration occurs.

## 3. Discussion

CAPV was a rare condition in the past, but with advances in imaging techniques, the number of patients with detected CAPV has increased. The age at diagnosis is variable and ranges from the prenatal period to late adulthood [10].

CAPV can cause a broad spectrum of clinical manifestations, which can result from concomitant congenital malformations or from subsequent syndromes.

Patients may be asymptomatic or present with features suggestive of a portosystemic shunt, such as hyperammonaemia, hypoalbuminemia, hypergalactosemia and mild liver function test abnormalities. This may result in persistent jaundice, growth failure, mental retardation or some stage of encephalopathy, chronic liver disease or an acute hepatic decompensation and hemorrhoidal bleeding [11].

CAPV is frequently combined with a benign or malignant hepatic neoplasm. Focal nodular hyperplasia is common, but an adenoma, hepatoblastoma or hepatocellular carcinoma may also occur [6,12]. A screening test for the early detection of liver tumours should be performed regularly in the follow-up of these patients. Our patients did not have a typical presentation of portal hypertension. They had minimal liver affection, without detected nodules or tumours.

Additionally, CAPV is often associated with genetic cardiovascular abnormalities (atrial septal defects, patent foramen ovale, ventricular septal defect, patent ductus arteriosus, dextrocardia), gastrointestinal abnormalities (polysplenia, biliary and duodenal atresia, choledochal cysts and intrahepatic gallbladders) or other skeletal and visceral anomalies. No associated congenital malformation was detected in our patients.

Significant morbidity and mortality may result from PoPH and hepatopulmonary syndrome. Although more publications dedicated to CPSS have been published lately, the vast majority are single case reports or cross-sectional studies without follow-up and a combination of patients with different types of CPSS [12,13,14,15]. There are only a few cases reporting on the direct association of PoPH with CAPV [16].

PoPH is a severe but well-known complication of portal hypertension among patients with or without underlying chronic liver disease. PoPH is defined as PAH (mPAP ≥ 20 mmHg, PCWP ≤ 15 mmHg and elevated PVR ≥ 3 WU) in patients with portal hypertension (portal pressure > 10 mm Hg), and it is a subset of group 1 pulmonary hypertension [17].

According to the published studies, PoPH occurs in 2–6% of patients with a pathologic or surgical portosystemic shunts [18]. On the other hand, in pulmonary hypertension registries, patients with PoPH represent 5 to 15% of patients with PAH.

Histologically, the pulmonary vasculature in patients with PoPH shows plexiform lesions, medial hypertrophy, intimal fibrosis, adventitial proliferation and evidence of microthrombi, similar to idiopathic PAH. While the hemodynamic profile of patients with PoPH is better than that of patients with idiopathic PAH, their overall mortality is comparable or worse [19].

The underlying mechanisms of the development of PoPH continue to be an area of active research. Postulated mechanisms of the development of PoPH include increased pulmonary blood flow and altered vascular compliance, leading to increased shear stress due to splanchnic vasodilatation and the formation of portosystemic shunts, which is a mechanical component.

As mentioned above, the presence of congenital portosystemic shunts, even in the absence of advanced liver disease and an increased portal pressure gradient, enable the shunting of the vasoactive substances by bypassing the liver metabolism and causing an imbalance between the pulmonary vasodilators and vasoconstrictors. The imbalance between these vasoactive substances in the pulmonary vasculature (including pulmonary vasodilators, nitric oxide and prostacyclin, as well as pulmonary vasoconstrictors endothelin-1, thromboxane A2 and serotonin) results in the injury of the pulmonary vessels, vasoconstriction and A subsequent increase in pulmonary vascular resistance [20].

Permanent vascular remodelling mediated through bone morphogenic protein 9 (BMP9), bone morphogenic protein receptor 2 (BMPR2) and Toll-like receptors (TLRs) activated by bacterial lipopolysaccharides (LSP) is under active research [21].

**Figure 8:**
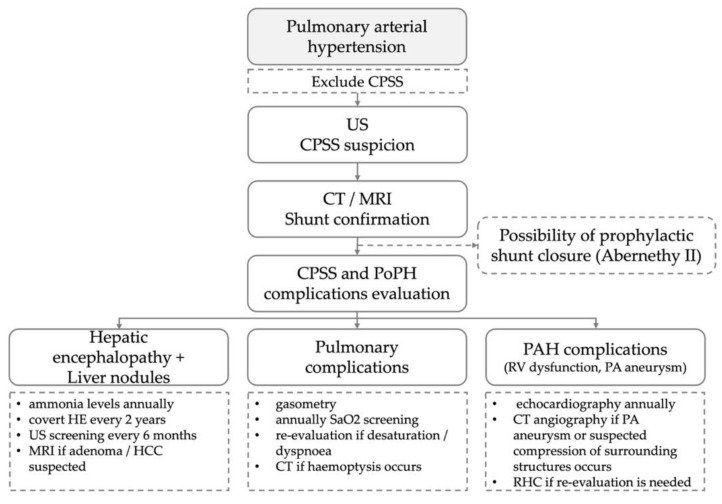
Algorithm for the diagnosis and follow-up of patients with congenital portosystemic shunts. CPSS—congenital portosystemic shunt; US—ultrasound; CT—computed tomography; MRI—magnetic resonance imaging; PoPH—portopulmonary hypertension; HE—hepatic encephalopathy; HCC—hepatocellular carcinoma; SaO2—oxygen saturation in arterial blood; PAH—pulmonary arterial hypertension; RV—right ventricle; PA—pulmonary artery; RHC—right heart catheterization.

In our work, we reported two cases of CAPV that presented as PAH without severe liver diseases, which were initially misdiagnosed as idiopathic PAH due to the lack of information on the aetiology and pathophysiology of PAH. Portal vein malformations and CPSS should be included and considered in the differential diagnosis of PAH, especially in paediatric or young patients. Here, we present an algorithm recommended for the diagnosis and follow-up of patients with CPSS and PAH (Figure 8).

Establishing the presence or absence of the intrahepatic vein is important when evaluating possible therapeutic options. The surgical or percutaneous closure of the shunt is possible only for Abernethy type II extrahepatic CPSS [22]. Unlike other types of CPSS, in CAPV, a correction of the anomaly in order to prevent the development of PoPH is usually not possible, but there are published cases where, even in these patients, after careful evaluation, hypoplastic intrahepatic portal veins were present, and this led to a malformation correction and shunt closure [7,22]. On the other hand, intervention can have potential pathophysiological consequences, such as the more frequent occurrence of hepatocellular carcinoma due to insufficient blood flow to the liver through the hypoplastic veins.

In cases of CAPV and PAH, liver transplantation is still considered as a primary option and should be accounted for early in the disease course so as to anticipate the development of severe PAH, pulmonary vascular remodelling and subsequent right ventricular dysfunction at the time of transplantation [15]. Our patients were not candidates for liver transplantation because of mild liver damage, with no possibility of the surgical correction of the shunts.

To prevent the progression of PAH, it is important to initiate an early and effective treatment with a specific pharmacotherapy for PAH that includes prostacycline analogues, phosphodiesterase-5 inhibitors and endothelin receptor antagonists [23].

In patients with already existing severe PAH, the regular evaluation of possible complications is crucial. PAH is an important cause of pulmonary artery dilatation, and this may be a reliable indicator of PAH. When the pulmonary artery diameter exceeds 40 mm, it is defined as a pulmonary artery aneurysm [24].

The extrinsic compression of LMCA by a pulmonary artery aneurysm is extremely rare but represents an important cause of sudden cardiac death in patients with PAH. In a recent study, a prevalence of 8.2% among patients with PAH was reported [25]. LMCA compression can exist without anginal symptoms and, therefore, computed tomography should be performed in patients at risk of it.

Aortocoronary bypass and unprotected LMCA stenting are the currently available revascularization strategies. However, in asymptomatic patients, as in our cases, no clear recommendations exist. Some authors have described an improvement in compression after the intensification of a specific PAH therapy [26].

Considering the high surgical risk in the context of severe PAH, LMCA stenting is usually favoured as the revascularization strategy of choice [27]. The largest cohort of 48 patients with PAH and angina symptoms due to extrinsic LMCA compression by pulmonary artery aneurysm were described by Galié et al. The symptoms of 45 patients were resolved by percutaneous coronary intervention (PCI), and 3 patients underwent surgical pulmonary artery reduction plasty. After the stenting procedure, 41 patients had improvement or showed a complete resolution of symptoms. Nine months after PCI, five patients had LMCA restenosis, and PCI was successfully repeated. The patients were followed-up over a mean of 4.5 years, and during that time, their survival was similar compared with a matched population with PAH and angina who did not have extrinsic compression of the LMCA [28]. In our patient, LMCA compression was not suitable for coronary stenting because of an unfavourable anatomy.

## 4. Conclusions

Portal vein malformations and CPSS should be included and considered in the differential diagnosis of PAH, especially in paediatric or young patients. Congenital portosystemic malformations represent an important and potentially treatable cause of PAH and, therefore, a careful assessment of the anatomy and testing of the portal vein hemodynamic should be performed before the possibility of a correction is declined. For CAPV, the options for corrective interventions are limited, and the patients are candidates for a liver transplant.

Even though recent years have seen the publishing of complex studies dedicated to CPSS, the natural course and prognosis of patients with CAPV remains unclear. The wide range of ages at diagnosis suggests that CAPV is not a fatal condition, and the comorbidities determine its course. A regular lifelong follow-up, including laboratory tests, image screening, echocardiography and the examination of respiratory functions, is recommended in order to provide the early detection and treatment of possible complications.

We define the PoPH as one of the major complications of CAPV, and once it is diagnosed, it is crucial for patients to undergo management and complex care in a specialized expert centre, where specific, targeted therapy can be administrated.

## Figures and Tables

**Table 1:** Right heart catheterization hemodynamic parameters (Patient 1).

RHC Parameters	Time of Diagnosis	Before Initiation of the Specific Therapy	8 Years of Specific Therapy
mPAP (mmHg)	59	90	49
PVR (WU)	8.1	12.6	6.9
PCWP (mmHg)	8	10	13
CO (L/min)	6.1	5.5	5.2

RHC—right heart catheterization; mPAP—mean pulmonary artery pressure; PVR—pulmonary vascular resistance; WU—Wood unit; PCWP—pulmonary capillary wedge pressure; CO—cardiac output.

**Table 2:** Functional and echocardiographic parameters during the follow-up period (Patient 1).

	Before ST	1 Year of ST	5 Years of ST	10 Years of ST
6MWT (m)	575	690	670	630
NYHA class	III	I-II	II	II
TR grad. (mmHg)	80	70	66	50
TAPSE (mm)	15	21	29	21
PA diameter (mm)	52	56	58	66

ST—specific therapy; 6MWT—six-minute walking test; NYHA—New York Heart Association; TR grad—tricuspid regurgitation gradient; TAPSE—tricuspid annular plane systolic excursion; PA—pulmonary artery.

**Table 3:** Right heart catheterization hemodynamic parameters (Patient 2).

RHC Parameters	Time of Diagnosis	Before Initiation of the Specific Therapy
mPAP (mmHg)	58	65
PVR (WU)	8.5	7.2
PCWP (mmHg)	9	2
CO (L/min)	5.8	8.7

RHC—right heart catheterization; mPAP—mean pulmonary artery pressure; PVR—pulmonary vascular resistance; WU—Wood unit; PCWP—pulmonary capillary wedge pressure; CO—cardiac output.

**Table 4:** Functional and echocardiographic parameters during the follow-up period (Patient 2).

	Before ST	1 Year of ST	5 Years of ST	10 Years of ST
6MWT (m)	560	710	708	640
NYHA class	II-III	I	I	II
TR grad. (mmHg)	65	55	50	67
TAPSE (mm)	27	29	25	28
PA diameter (mm)	42	45	52	53

ST—specific therapy; 6MWT—six-minute walking test; NYHA—New York Heart Association; TR grad—tricuspid regurgitation gradient; TAPSE—tricuspid annular plane systolic excursion; PA—pulmonary artery.

## Data Availability

The data underlying this article are available in the article.
